# When Aromaticity Falls Short in Molecule–Surface Interactions

**DOI:** 10.1021/acs.jpcc.5c05441

**Published:** 2025-09-26

**Authors:** Jonas Brandhoff, Richard K. Berger, Felix Otto, Maximilian Schaal, Lorenz Brill, Oliver T. Hofmann, Peter Puschnig, Torsten Fritz, Roman Forker

**Affiliations:** † Institute of Solid State Physics, 9378 Friedrich Schiller University Jena, Helmholtzweg 5, Jena 07743, Germany; ‡ Institute of Solid State Physics, 27253 Graz University of Technology, Petersgasse 16, Graz 8010, Austria; § Institute of Physics, University of Graz, Universitätsplatz 5, Graz 8010, Austria

## Abstract

Aromaticity is one of the most important concepts in organic chemistry. There are cases in which a molecule undergoes changes to increase its aromaticity. This higher aromaticity comes with an energetic gain and is commonly referred to as aromatic stabilization. Previously, it has been reported that some molecules undergo such a stabilization when adsorbing on a surface, which has been identified as the reason for charge transfer into the molecular π-system. Utilizing photoemission orbital tomography and density functional theory, we investigate changes in the molecular π-system upon adsorption and elucidate the influence on the aromaticity. We demonstrate how the energetic gain from an aromatic stabilization on surfaces can be outweighed by hybridization. Uncovering a mechanism in which the molecular π-system forms dative bonds with the surface, our study reveals that the concept of aromatic stabilization on surfaces has been incomplete so far.

## Graphical abstract

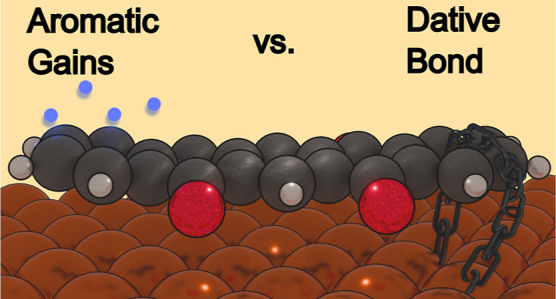

## Introduction

Aromaticity has proven to be an important concept in chemistry.^1,2^ It describes the fact that certain electronic configurations—typically cyclic conjugated hydrocarbons with 4*n* + 2 π-electrons^3,4^ —exhibit favorable resonance structures, leading to substantial energetic stabilization. In chemical design, this aromatic stabilization is often exploited to induce charge separation between the donor and the acceptor part of a molecule, e.g., in the field of nonlinear optics.^5–7^ Also, for metal–organic interfaces, the so-called surface-induced aromatic stabilization has been identified as a strong driving force for charge transfer.^8,9^

In contrast, here we report about systems which, despite showing charge transfer, exhibit an electron configuration which is far away from maximizing aromaticity. This implies that there is a driving force beyond aromatic stabilization at interfaces. Our joint computational and experimental investigation demonstrates this mechanism and scrutinizes several cases where it is dominant. Investigating the nature of bonds present between substrate and adsorbate, we find that charge transfer in these systems is not only of ionic nature, but dominantly mediated by dative bonds between formerly unoccupied π-orbitals and the surface. As these different bond archetypes affect the interplay between the adsorption geometry, packing density, and the electronic structure of the adsorbed molecules in a fundamentally different way, this directly guides the future design of charge transfer interfaces.

## Methods

### Sample Preparation

5,7,12,14-Pentacenetetrone (P4O, CAS registry no. 23912-79-0) was purchased from Alfa Aesar and purified using temperature gradient sublimation. The Cu(111) and Ag(111) single crystals were bought from MaTecK with a nominal purity of 99.999% and prepared using subsequent sputtering (±45°, 700 eV, 4 μA/cm^2^ for 30 min) and annealing steps (600 °C for 30 min). P4O was deposited from a crucible at 200 °C onto the Cu(111) and Ag(111) substrates kept at room temperature. The structures were checked using distortion-corrected low-energy electron diffraction (LEED) and scanning tunneling microscopy (STM).

### Computational Details

Density functional theory (DFT) calculations were performed using the FHI-aims code^10^ using the PBE exchange correlation potential^11^ and vdW^TS^ correction parametrized for metal surfaces.^12^ We used “tight” basis set defaults, as shipped with the code. All calculations were converged until the total energy and electron density reached thresholds of 1 × 10^–6^ eV and 1 × 10^–5^ e Å^–3^, respectively. To perform geometry optimizations, we relaxed the atomic positions until the remaining forces fell below 0.01 eV/Å on each atom. We sampled the Brillouin zone with 10 × 6 × 1 *k*-points for Cu(111) and 9 × 9 × 1 *k*-points for Ag(111). The surface was modeled as a slab of five layers for Cu(111) (four layers for Ag(111)), where the top two layers were allowed to relax during optimization. A dipole correction was used to electrostatically decouple the periodic replica in *z*-direction.^13^ We used an energetical broadening of 0.1 eV in our DFT calculations. Thus, a Gaussian with a standard deviation of 0.1 eV would be the sharpest peak in the density of states. Full momentum maps were simulated by approximating the photoemission final state by a plane wave as described in ref 14, where the initial state Bloch functions have been obtained by utilizing the VASP code.^15–17^ These calculations used the converged geometries of our FHI-aims calculations. A comparison for the resulting maps and the molecular orbital projected density of states (MOPDOS) can be found in Supporting Information Section S4. The real-space geometry of the orbitals are visualized using VESTA.^18^ Simulation of the STM image was done using WSxM 5.0.^19^

### Experimental Details

The lattice used in the DFT calculations was determined by distortion-corrected LEED^20^ in conjunction with STM. STM was done at 4.5 K using a Joule–Thomson (JT)-STM (Specs Surface Nano Analysis) with a Pt–Ir wire tip. Photoemission orbital tomography (POT) was done with monochromatized and p-polarized He Iα emission (Specs UVLS with TMM 304). The photoelectrons were detected using a Specs PHOIBOS 150 hemispherical analyzer equipped with a delay-line detector (3D DLD4040-150). To use consistent signs between experiment and theory, we plot the binding energies of the occupied states as *E* – *E*_F_, therefore with a negative sign. The fitting of the experimental data to the Fourier transform of the free molecular orbitals was done using an implementation of the k-map.py code.^21^ Fitting was performed using the python package lmfit.^22^

## Results and Discussion

### Highly Ordered Films of P4O on Cu(111)

A prototypical system that should exhibit surface-induced aromatic stabilization is 5,7,12,14-pentacenetetrone (P4O) on Cu(111).^8^ However, it has been previously shown that this stabilization is incomplete, and it was argued that additional effects uncaptured by the mechanism of surface-induced aromatic stabilization play a role.^23,24^ Therefore, we choose this as the primary system for our investigation. In the gas phase, P4O is not fully π-conjugated. Rather, it is more accurately described as three benzene moieties connected via two carbonyl groups each. This is illustrated using the Clar structure^25^ in Figure 1a. It has been claimed that upon charge transfer from the Cu(111) surface, the π-system delocalizes over the entire carbon backbone of the molecule and, thus, P4O becomes as aromatic as pentacene (PEN, CAS registry no. 135-48-8) in the gas phase.^8^ Because for organic films on metals the degree of charge transfer may depend on the packing density^26^ or the relative adsorption sites and orientation of the molecules,^27,28^ it is helpful to first determine the structure of P4O on Cu(111).

**Figure 1:**
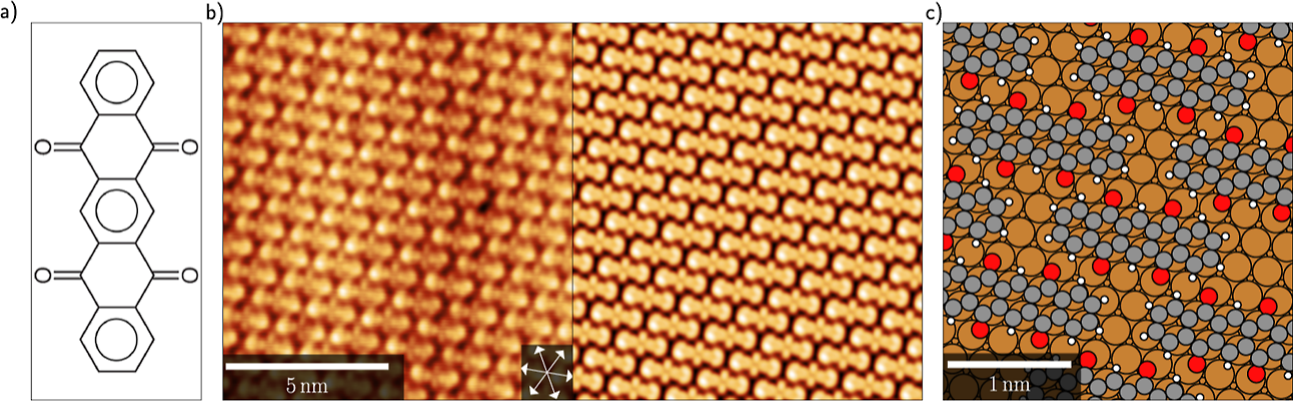
(a) Clar structure of P4O. (b) STM image of the first P4O layer on Cu(111) (left), *V*_bias_ = +0.1 V (unoccupied states), *I* = 170 pA, along with a simulated STM image with the same scale and bias voltage (right). (c) DFT-calculated structure of P4O on Cu(111). The oxygen atoms (red) bend down toward the Cu (dark orange) surface, and the ends of the carbon (gray) backbone bend upward. The centers of the carbon rings are located above a bridge position resulting in equivalent adsorption sites of the oxygen atoms.

An STM image is shown in Figure 1b left. LEED experiments (see Supporting Information, Section S1) confirm this structure and indicate long-range ordering of the monolayer. DFT calculations based on this unit cell confirm the STM contrast (see Figure 1b right). The fully optimized DFT geometry places the center of the molecule over a bridge site (shown in Figure 1c) and at an (averaged) adsorption height of 2.24 Å above the topmost Cu atom, with the oxygen atoms 19 pm below and the ends of the molecule 15 pm above the average carbon plane. Both adsorption height and bending are consistent with previously conducted X-ray standing waves experiments.^8^ The small adsorption distance, which barely exceeds the sum of the covalent radii of Cu and C, is already a first indication for a strong interaction between Cu(111) and P4O.^29,30^

### Electronic Configuration

To understand the electronic structure of this interface, we examine its MOPDOS. The MOPDOS reveals the energetic positioning of the molecular states upon adsorption. Furthermore, the integral for a specific state up to the Fermi level yields the corresponding occupation. Additional insight is gained from the molecular orbital overlap population (MOOP^31–33^ ). The MOOP scales the DOS by the coefficients of the involved metal and molecule states as well as their overlap. In the MOOP, positive values indicate bonding and negative values antibonding contributions to the bond. Conjointly, MOPDOS and MOOP provide a powerful framework to understand the details of the interaction between a molecule and a surface. As a crucial foundation for the interpretation of our results, we will briefly discuss different bond archetypes and how they can be identified using the MOPDOS and MOOP.

**Covalent bonds** are formed when both bonding partners have orbitals at similar energies and the wave function overlap between them is large. It leads to the formation of a bonding (=stabilized) and an antibonding (=destabilized) hybrid orbital. On the surface, where individual adsorbate orbitals interact with a metal band, this is reflected either by a broadened hybrid band (for weak interaction) or a splitting into a band with multiple peaks (for strong interaction).^34,35^ Typically, covalent bonds occur when both bonding partners contribute electrons. In the MOOP, covalent bonds can be identified by their high absolute value (due to high overlap and close to identical coefficients for the involved orbitals of both partners). Additionally, the MOPDOS shows broadened states or, possibly, a multiple-peak structure.

A special case of covalent bonds are **dative bonds**. These occur when one of the initial orbitals is filled and the other is empty. Dative bonds behave similarly to covalent bonds and are dependent on the wave function overlap, but with the qualitative difference that if the coefficients of the involved orbitals are similar on both bonding partners, a nominal charge transfer occurs. A joint examination of the MOPDOS and the MOOP allows to classify the bond: If all aforementioned properties of the covalent bond are identified for a formerly unoccupied orbital together with a net charge transfer into the corresponding hybrid orbital, a dative bond is present.

Finally, **ionic bonds** are triggered by the equilibration of the chemical potential of both bonding partners. In an orbital picture, it occurs when an empty orbital of one bonding partner is energetically lower than the filled orbital of the other. Then, energy is gained by transferring the electron to the lower-energy orbital. Archetypical ionic bonds are independent of the wave function overlap. Ionic interactions manifest as small contributions in the MOOP and preserve a sharp, single-peak structure in the MOPDOS.

A detailed discussion of these bond archetypes and how they are reflected in the MOOP and MOPDOS can be found in the Supporting Information, Section S2. Note that in reality, bonds generally are not purely ionic or covalent, but fall in-between. When MOPDOS and MOOP are examined together, insights into the dominant contribution are gained, which does not exclude other minor contributions. In the following we will use these considerations to understand the interactions between P4O and the Cu(111) surface, for which the results are shown in Figure 2.

**Figure 2:**
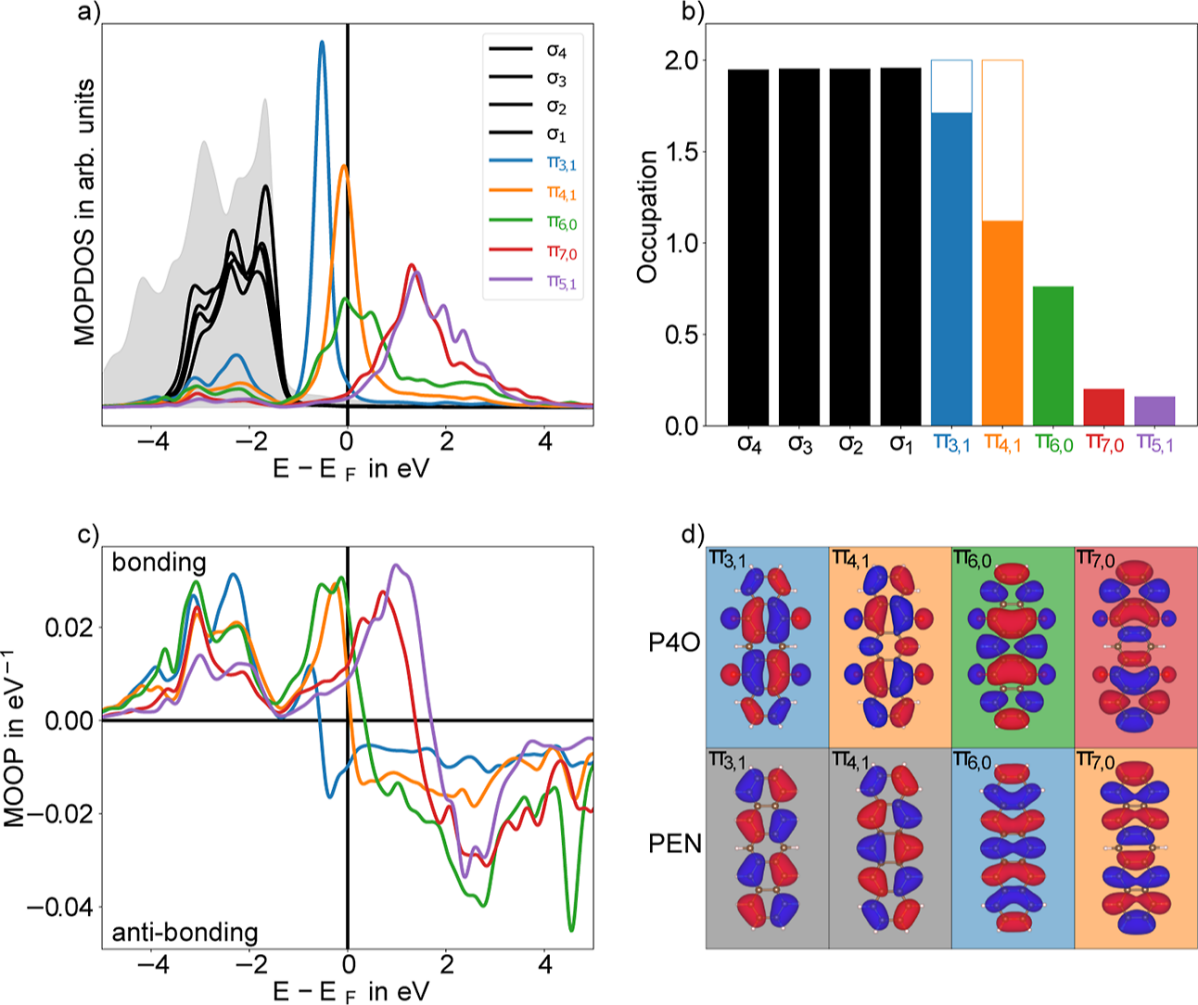
(a) Molecular orbital projected DOS (MOPDOS) around the Fermi level. The orbitals which are fully occupied for P4O in the gas phase are shown in black, those unoccupied in the gas phase are in color. The π_4,1_ and π_6,0_ orbitals are energetically around the Fermi level and both are partially occupied. The π_3,1_ orbital is almost fully occupied and shifted away from the Fermi level. In gray the density of the d-orbitals of the substrate is shown (not to scale). (b) Corresponding occupations of the orbitals shown in (a). The outlined white parts correspond to the occupations of PEN in the gas phase. (c) The molecular orbital overlap population (MOOP) for the relevant π-orbitals. Positive (negative) values correspond to bonding (antibonding) contributions. All π-orbitals shown exhibit net bonding character to the surface. (d) Real-space representations of the frontier π-orbitals with their respective labels. A comparison to the gas-phase PEN molecule is shown. The colors represent the gas-phase ordering of orbitals (blue corresponds to the LUMO, orange to the LUMO + 1, and so on).

To enable a better comparison between different molecules, the π-orbitals are labeled by the number of nodal planes parallel to the short and long molecular axes, separated by a comma, as suggested by Haags et al.^36,37^ We note in passing that in the gas phase the four highest occupied molecular orbitals of P4O are nonbonding oxygen lone-pair orbitals. These do not impact the system’s aromaticity directly, which is defined only via its π-system.

Figure 2b shows the occupations of the molecular frontier orbitals upon adsorption on the Cu(111) surface. We find a large nominal charge transfer on the Cu(111) surface, with the π-system of P4O receiving approximately four electrons. These occupy mainly three formerly unoccupied levels, namely the π_3,1_, π_4,1_, and π_6,0_-orbitals (i.e., the former LUMO, LUMO + 1, and LUMO + 2, see Figure 2). For all of these contributions, we find a substantial broadening and even a multipeak structure in the MOPDOS (with contributions near the Fermi level and a smaller peak between −4 eV and −2 eV, i.e., in resonance with the Cu d-bands, Figure 2a). The distance sweep (which will be discussed in more detail later) in the Supporting Information Figure S12 shows how this substantial broadening is caused by the molecule–surface interaction and furthermore gives a reference for the MOPDOS far away from the surface. Additionally, the MOOP shows large bonding values for the corresponding states (Figure 2c). Conversely, orbitals which show close to zero MOOP while maintaining a sharp, well-defined single peak in the MOPDOS—which would be indicative of a prototypical ionic charge transfer—are not found in our analysis. Evidently, the observed charge transfer stems from a hybridization of the formerly unoccupied π-orbitals of the molecule with the surface, forming a dative bond. This is further supported by investigating the overlap populations between individual atoms (see Supporting Information, Section S3), showing bonds between not just the oxygen atoms and the surface but also between the carbon atoms and the surface.

To ensure that both amount and nature of the charge transfer are not methodological artifacts (after all, PBE-based calculations are known to yield incorrect orbital positions and potentially spurious charge transfer,^38–41^ ) we corroborate the results with photoemission orbital tomography (POT^42^). POT has proven to be a viable tool to investigate the aromaticity of adsorbed molecules.^43^ Photoelectron momentum maps for orbitals just below the Fermi level (i.e., at binding energies of −0.73 eV and −0.13 eV) are shown in Figure 3a,b. To identify the nature of the involved molecular orbitals, a simple Fourier transform of the gas-phase molecular orbitals, following the plane-wave final state approximation, was performed and compared to the measured momentum maps. Furthermore, we determine momentum maps by a complete simulation of the *k*-space intensity distribution using the entire system and find consistent results (see Supporting Information, Section S4).

**Figure 3:**
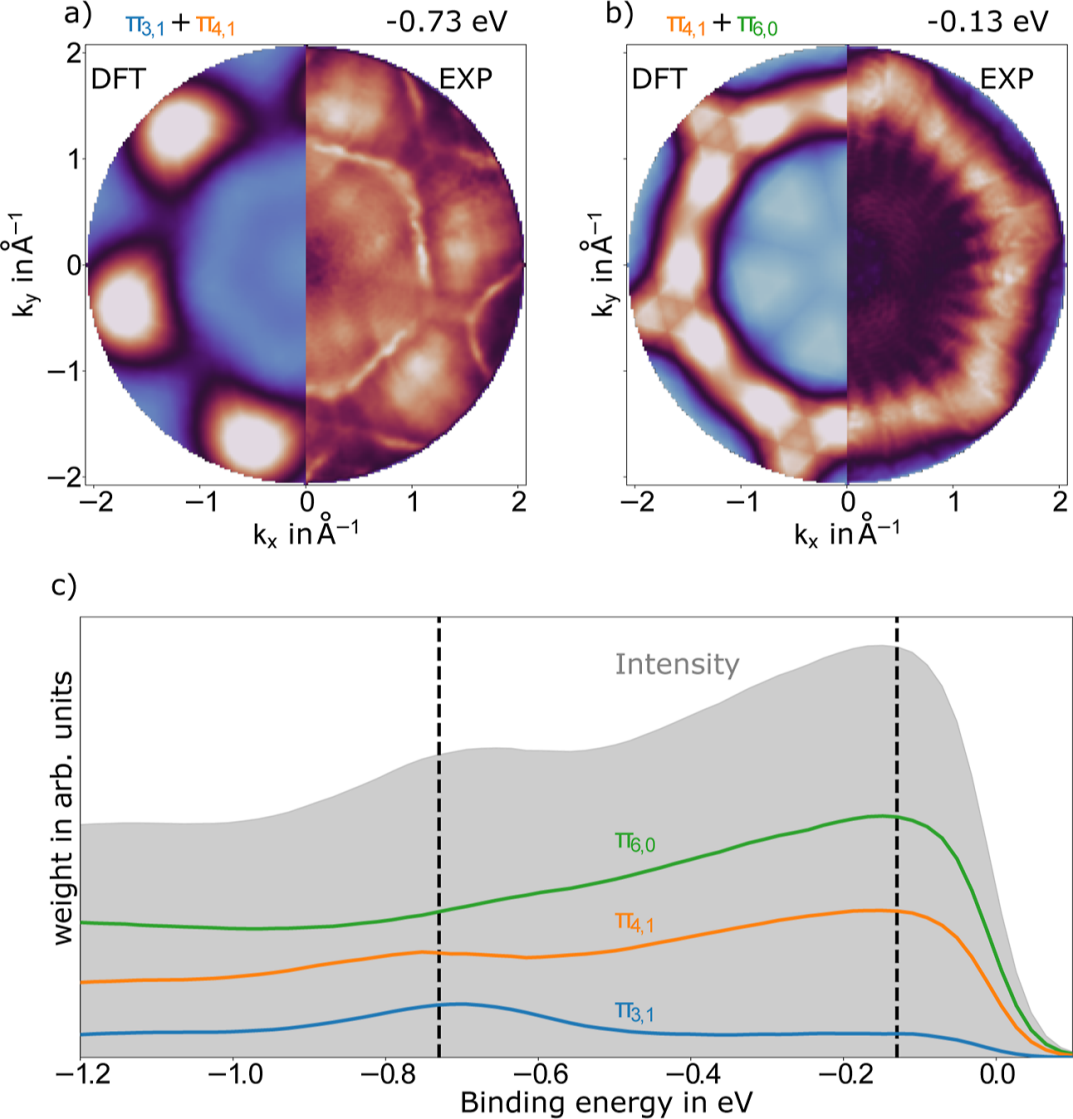
Simulated and measured photoelectron momentum maps of P4O on Cu(111). The left half of each map is the simulation. The corresponding right half shows the measured intensity distribution. In all cases the simulation is scaled to the intensity maximum of the measurement, and the substrate symmetries are taken into account. The simulations of (a,b) are done using the gas-phase P4O molecule. Map (a) is taken at a binding energy of −0.73 eV and corresponds to the intensity distribution of the π_3,1_ and π_4,1_ orbital emissions; (b) is taken at a binding energy of −0.13 eV and shows emission from the π_4,1_ and π_6,0_ orbitals. A full simulation of the *k*-space can be found in the Supporting Information, Section S4. (c) Experimentally obtained, energy-dependent orbital deconvolution. The weight for each π-orbital is shown in the corresponding color and the *k*-integrated intensity is shown as gray shaded background. The dashed black lines indicate the energetic positions of the momentum maps shown in (a,b).

The momentum maps confirm that all three of the initially unoccupied orbitals (π_3,1_, π_4,1_, and π_6,0_) are found to be at least partially below the Fermi level and, thus, (partially) occupied, as shown by the energy-dependent deconvolution of the π_3,1_, π_4,1_, and π_6,0_-orbitals in Figure 3c. It reveals that the π_4,1_ and π_6,0_-orbitals have their largest contribution at the Fermi level and that the π_3,1_-orbital has the highest contribution further away at around −0.73 eV. All of these results are consistent with the above-discussed theoretical results.

Further, the question arises how these dative bonds affect the aromaticity of the adsorbed molecule. For this we define a “similarity”-measure *A*_sim_ with reference to PEN in the gas phase. A detailed discussion about this measure is provided in the Supporting Information, Section S5. In short, it can be seen as a scale of how similar the molecular π-system and the π-system of gas-phase PEN are. A value of *A*_sim_ = 1 would correspond to an equivalent π-system of both molecules, i.e., the adsorbate reaching the reference aromaticity of PEN. This would be the case if the four electrons received from the substrate would occupy the π_3,1_ and π_4,1_ orbitals (and none beyond, compare Figure 2d). However, for P4O on Cu(111) this is not the case. Instead, the observed configuration qualitatively resembles that of PENs first excited state, which is less aromatic than PEN in its ground-state.^44,45^ In fact, for P4O on Cu(111) we find a value of *A*_sim_ = 0.44 for the similarity to the reference aromaticity, indicating an incomplete aromatic stabilization.

This deviation from the reference aromaticity sets the stage for further questions: first, if the electron number is correct to allow for higher aromaticity, why is it not realized for P4O on Cu(111)? Would a different adsorption height allow for a more PEN-like aromatic structure, and if yes, why is that (apparently) not the energetically most favorable configuration? Second, is P4O on Cu(111) a special case, or is it representative of a wider class of interfaces? To answer these questions, we first turn to the adsorption of P4O on a different metal, namely Ag(111).

### P4O on Ag(111) and Comparison to Cu(111)

As shown above, when P4O adsorbs on Cu(111) the aromaticity increases, but the electronic configuration of gas-phase PEN is not reached. This discrepancy mainly stems from the fact that the π_4,1_-orbital of P4O (which in gas-phase PEN would be the HOMO) does not become fully occupied, while higher orbitals do become partially occupied as well. The question arises whether a less reactive metal surface like Ag(111) results in a charge transfer only into the π_3,1_ and π_4,1_ orbitals.

In Figure 4a,c the MOPDOS and MOOP for P4O on Ag(111) are shown, along with the resulting occupations in (b). Several striking differences to the adsorption on Cu are obvious: The molecular states are much less broadened and essentially exhibit single-peak character, indicating a much weaker interaction. The MOOP associated with the π_3,1_-orbital is large with small additional bonding contributions at −3 eV, indicating that again dative bonds, rather than purely ionic interactions, dominate the interaction between the molecular states and the surface. Furthermore, the π_3,1_-orbital is energetically located at the Fermi level, rather than mostly below it, and all higher-lying orbitals are (almost) completely above the Fermi level. Consequently, the π-system only receives roughly one electron—much too little to reach the aromatic degree of gas-phase PEN, which would require four electrons (compare Figure 2d). Using POT we can verify this electronic configuration of P4O on Ag(111) (see Supporting Information, Section S7). Thus, on both substrates, P4O does not maximize its potential for aromatic stabilization, albeit for different reasons: on Cu(111), the charge transfer is large enough, but the electronic configuration is incorrect, while on Ag(111), the charge transfer is too small. The question arises what the cause of this large difference between the Cu(111) and Ag(111) surface is.

**Figure 4:**
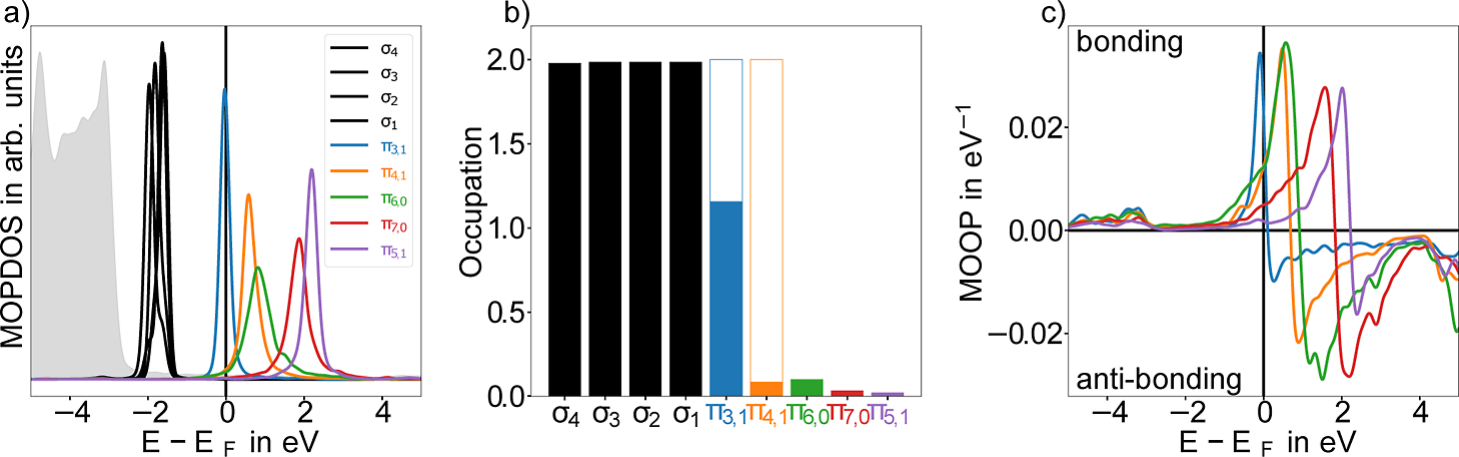
(a) MOPDOS for P4O on Ag(111). In gray the density of the d-orbitals of the substrate is shown (not to scale). (b) The corresponding occupation for each state shown in (a); the outlined bars correspond to the occupation of PEN in the gas phase. Thus, a complete aromatic stabilization would have filled these bars entirely and the aromaticity similarity *A*_sim_ would be 1. (c) MOOP for P4O on Ag(111). Compared to the Cu(111) system the bonding contributions toward the substrate are much smaller.

An important difference between both substrates is the work function, which is 4.86 eV for Cu(111) and 4.45 eV for Ag(111) (both obtained from DFT). Because of the smaller work function, one could expect a larger charge transfer on Ag(111). However, this is not the case since the interaction is dominated by the creation of dative bonds. In fact, we find the exact opposite with a lower amount of charge transfer compared to Cu(111). Only for distances further away from the surface this behavior reverses as the ionic interactions gain in importance, see Figure 5. This hypothetical distance dependence yields relevant insights into the physics of the system and will be elucidated in the following.

**Figure 5:**
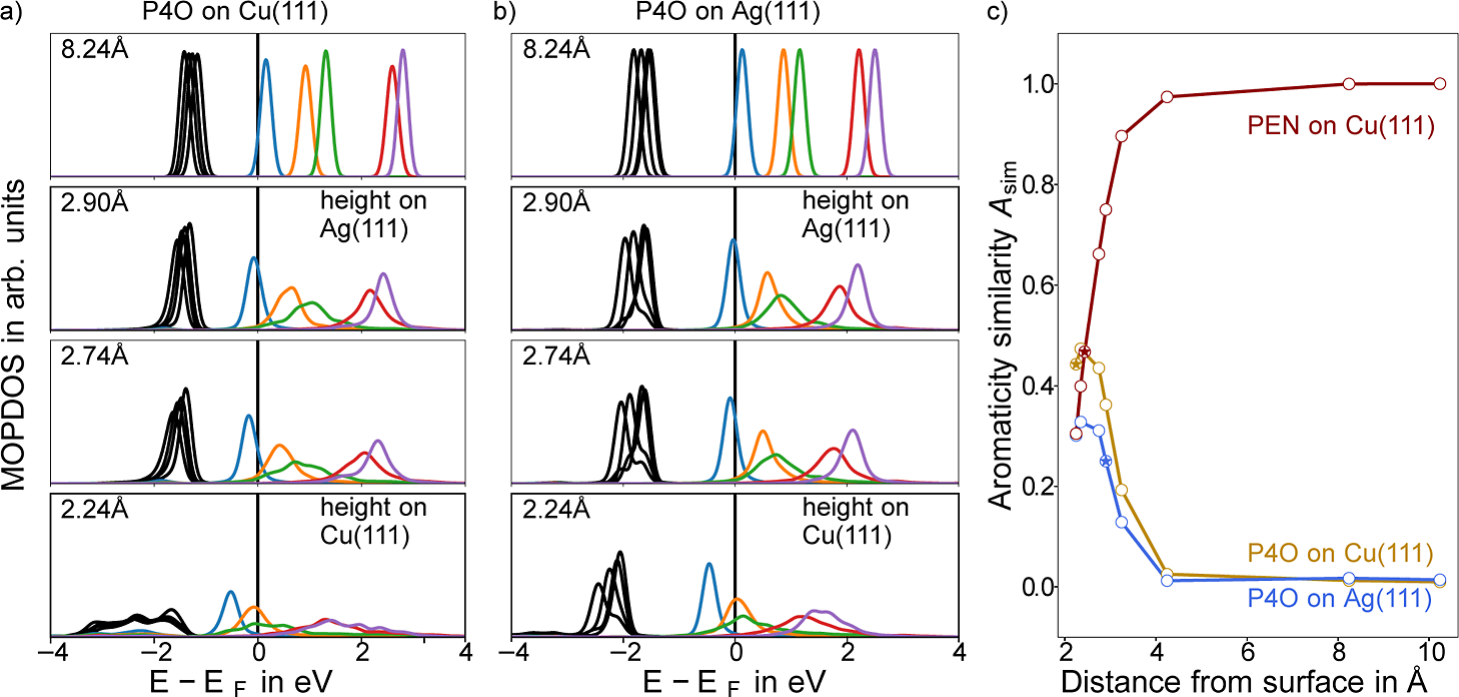
MOPDOS for different distances from the Cu(111) (a) and Ag(111) (b) surface. The distance is defined as the difference between the average carbon height and the average height of the topmost metal atoms. The adsorption heights for P4O on Cu(111) and Ag(111) are annotated accordingly. For the same distances both systems show qualitatively the same behavior. A full sweep for all investigated distances can be found in the Supporting Information, Section S8. (c) The similarity to the reference aromaticity (with respect to PEN in the gas phase) for P4O on Cu(111) (dark yellow) and on Ag(111) (blue), as well as PEN on Cu(111) (dark red) depending on the distance from the surface. The star-filled points correspond to the energetic minimum of each system.

A key factor governing hybridization is the overlap between the molecular states and the metal states, which depends exponentially on the distance from the surface. On Cu(111) the average carbon plane of P4O is 2.24 Å above the average Cu plane of the topmost layer, while on Ag(111) the average distance is 2.90 Å. To quantify the impact of the different adsorption distances on the charge transfer and the emerging aromatic stabilization, we show the MOPDOS for a set of different hypothetical adsorption heights in Figure 5a,b. It reveals that, for the same adsorption distances, there is no significant difference between P4O on Ag(111) and on Cu(111). Furthermore, Figure 5c demonstrates how different hypothetical adsorption heights affect the aromaticity similarity to PEN.

We find that at large distances, almost no charge transfer between the metals and P4O occurs, leaving the molecule nonaromatic. As the molecule approaches the surface, the aromaticity similarity *A*_sim_ increases exponentially. If the interaction with the surface would be of purely ionic nature, the charge transfer, and consequently the aromaticity similarity, should change with one over distance (explained in the Supporting Information, Section S6). Turning to the system PEN on Cu(111), the aromaticity similarity decreases as it approaches the surface. We also find the emergence of dative bonds for PEN on Cu(111) with the connected charge transfer decreasing the similarity to the reference aromaticity (see Supporting Information, Section S9).

Most interestingly, however, the adsorption height of P4O does not coincide with the highest similarity to the reference aromaticity. Rather, on Ag(111) the molecule remains too far away from the surface, while on Cu(111), it is too close. This is likely due to the fact that the energetic gain realized by approaching pentacene’s degree of aromaticity is only one of many factors in these strongly hybridized systems. In fact, we argue that in the case of strong dative bonds the higher aromaticity on the surface for these systems can be viewed as a necessary byproduct of the hybridization, rather than the inherent cause of the charge transfer.

To assess whether the behavior of P4O on coinage metals represents a special case, we investigated several additional systems, where the aromaticity in the gas phase is impeded by carbonyl groups. The results, summarized in the Supporting Information, Section S10, consistently demonstrate that for this class of adsorbates, the formation of dative bonds dominates over ionic-type charge transfer. This indicates that dative bonds, in addition to ionic charge transfer, play a substantial role at metal–organic interfaces.

## Conclusion

We investigated molecule–substrate systems which have been reported before to show aromatic stabilization on surfaces. Previously, this aromatic stabilization has been considered as the main driving force for charge transfer from the substrate into the molecule. In this study we have uncovered an additional mechanism which involves the creation of dative bonds between formerly unoccupied molecular π-orbitals and the substrate. Investigating P4O on Cu(111) in detail, we found that even though a large charge transfer is caused by the bond formation, the distribution of charge does not match the electronic structure of gas-phase PEN. Hence, the degree of aromaticity of gas-phase PEN is not reached for P4O on Cu(111), demonstrating that aromatic gains can be outweighed by the bond formation. Moving to silver, we found a much weaker interaction between P4O and Ag(111), resulting in a less pronounced charge transfer. We elucidated the difference between the systems, showing that the work function plays a subordinate role here.

Finally, we argue that in these systems charge transfer in the ionic sense plays a secondary role and that the aromatic gains are a byproduct of the strong hybridization. This behavior is found for several molecule–substrate systems demonstrating the importance of this additional mechanism. We suggest that the relative importance of ionic and covalent charge transfer strongly depends on the details of the system, specifically the adsorption distance. While for molecules which are tightly bonded to the surface, such as it is the case for the systems investigated in this study, the large wave function overlap practically enforces dative bonds, for molecules which adopt larger adsorption distances, such as F_4_TCNQ^46^ or F_6_TCNNQ,^47^ the ionic character of the interaction becomes increasingly pronounced. Systems that exhibit bistable adsorption, in which a molecule can be switched between a physisorbed and a chemisorbed state, would be intriguing model systems for further investigations. These systems would allow to systematically tune the aromaticity and the dominant interactions with the surface.^48–50^ Moreover, they would make it possible to experimentally observe the distance dependence without changing the surface, thereby eliminating other factors that may influence the aromaticity.

## Supplementary Material



## Notes

Distortion-corrected LEED of P4O on Cu(111); bond types, polarity, and charge transfer; atomic overlap population; complete simulation of the photoelectron momentum map; similarity measure of aromaticity; distance dependence; experimental data of P4O on Ag(111); distance sweeps for P4O on Cu(111) and on Ag(111); PEN on Cu(111); further investigated systems (PDF)
