# Supplementary material for: When Aromaticity Falls Short in Molecule–Surface Interactions
Source: J Phys Chem C Nanomater Interfaces. 2025 Sep 26;129(46):20738–45. doi: 10.1021/acs.jpcc.5c05441 (PMC12641464; doi:10.1021/acs.jpcc.5c05441)
Supplement: Supplementary file 1 [file jp5c05441_si_001.pdf]

Supporting Information: When Aromaticity Falls Short in Molecule–Surface Interactions

Jonas Brandhoff,† Richard K. Berger,‡ Felix Otto,† Maximilian Schaal,† Lorenz Brill,† Oliver T. Hofmann,*,‡ Peter Puschnig,¶ Torsten Fritz,† and Roman Forker*,†

†*Institute of Solid State Physics, Friedrich Schiller University Jena, Helmholtzweg 5, Jena, 07743, Germany*

‡*Institute of Solid State Physics, Graz University of Technology, Petersgasse 16, Graz, 8010, Austria*

¶*Institute of Physics, University of Graz, Universitätsplatz 5, Graz, 8010, Austria*

E-mail: o.hofmann@tugraz.at; roman.forker@uni-jena.de

S1 Distortion-corrected LEED of P4O on Cu(111)

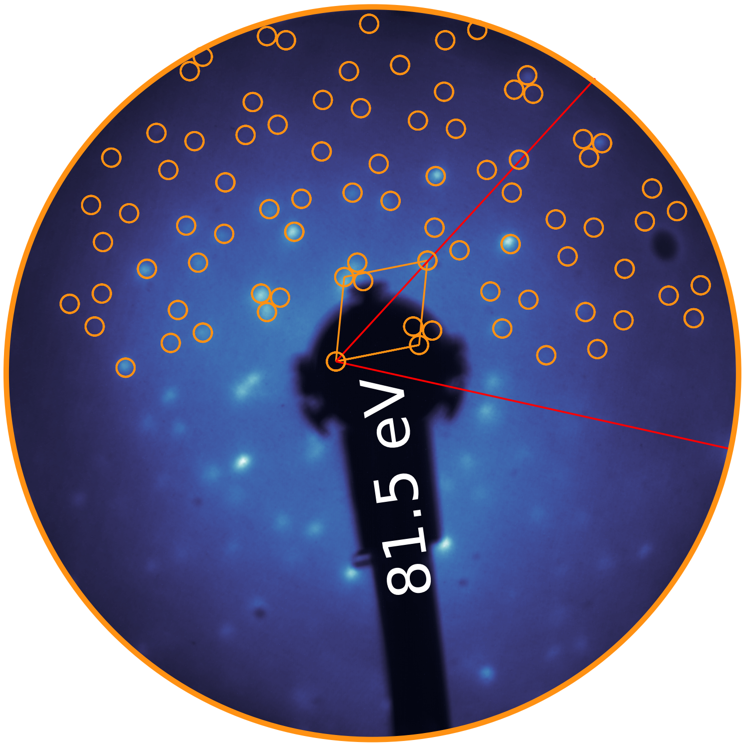

Figure S1: Distortion-corrected LEED image of a P4O monolayer on Cu(111) taken at an energy of 81.5 eV. The LEED simulation of the fitted structure is shown in orange (P4O) and red (Cu(111)). The epitaxial registry is commensurate and is used for the DFT calculations.

Using a fitting procedure we find a commensurate epitaxial registry with the epitaxial matrix given as

$$1.00(1) \cdot \begin{pmatrix} 5.00(1) & 3.00(1) \\ -2.00(1) & 3.00(1) \end{pmatrix}.$$

The lattice vectors of the adsorbate can thus be expressed as: $\vec{a}_1 = 5\vec{s}_1 + 3\vec{s}_2$ and $\vec{a}_2 = -2\vec{s}_1 + 3\vec{s}_2$. Here, the substrate lattice at room temperature is given by $|\vec{s}_1| = |\vec{s}_2| = 2.5560$ Å and $\measuredangle(\vec{s}_1, \vec{s}_2) = 120°$.[S1]

S2 Bond types, polarity, and charge transfer

A simple yet powerful model to describe the bond between two components A and B is to write the bond state $|\Psi\rangle$ as a linear combination of only two states $|\Phi_A\rangle$ and $|\Phi_B\rangle$ that represent the contributions of the components A and B, according to Eqn. (1). A and B can be two atoms that form a bond via the hybridisation of their atomic orbitals. However, considering an adsorption process, A and B can also refer to a molecule and a substrate that form a bond via the hybridisation of molecular orbitals and surface states of the substrate.

$$|\Psi\rangle = c_A|\Phi_A\rangle + c_B|\Phi_B\rangle \tag{1}$$

The simple form of Eqn. (1) allows for the definition of the polarity according to Eqn. (2)

$$p = |c_A|^2 - |c_B|^2 \tag{2}$$

Using the bond model according to Eqn. (1) allows for the classification of different bond types. First, a covalent bond is characterized by similar contributions of $|\Phi_A\rangle$ and $|\Phi_B\rangle$ to the bond state $|\Psi\rangle$, i.e., $|c_A|$ and $|c_B|$ are similar. This is typically the case when $|\Phi_A\rangle$ and $|\Phi_B\rangle$ are similar in energy (i.e., $\epsilon_A = \epsilon_B$) and have a significant overlap S_{AB}. In that case, a proper linear combination of $|\Phi_A\rangle$ and $|\Phi_B\rangle$ yields a bonding state $|\Psi\rangle$ with an energy E_{bond} that is reduced compared to the energies ϵ_A and ϵ_B, of the single orbitals $|\Phi_A\rangle$ and $|\Phi_B\rangle$, as shown in Fig. S2. The energy E_{bond} of the bond state $|\Psi\rangle$ decreases with the overlap S_{AB} and increases with the energy difference $V_p = |\epsilon_A - \epsilon_B|$ between the states $|\Phi_A\rangle$ and $|\Phi_B\rangle$.

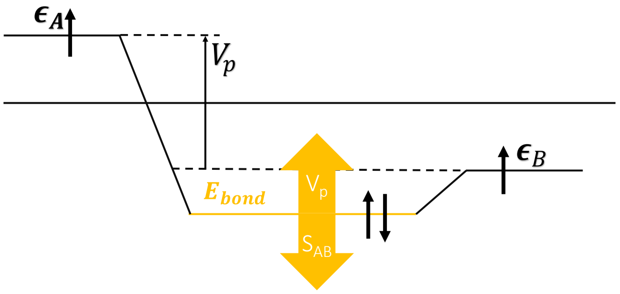

Figure S2: Energy and occupation of a typical covalent bond state $|\Psi\rangle$ for $S_{AB} > 0$ depending on the energy difference V_p and the overlap S_{AB} of the initial states $|\Phi_A\rangle$ and $|\Phi_B\rangle$.

Since for covalent bonds the contributions $|c_A|$ and $|c_B|$ are similar, the polarity p is small. The most common case of a covalent bond is the case in which the orbitals $|\Phi_A\rangle$ and $|\Phi_B\rangle$ are initially occupied by one electron each and the system can reduce its energy by occupying the bond state $|\Psi\rangle$ with both of these electrons, i.e. both components A and B contribute one electron to the covalent bond state $|\Psi\rangle$. Therefore, in the case of an ordinary covalent bond, there is negligible charge transfer between the components A and B.

A special case of a covalent bond is the dative bond. In this case, again a proper linear combination of $|\Phi_A\rangle$ and $|\Phi_B\rangle$ yields a bonding state $|\Psi\rangle$ that has a reduced energy E_{bond} compared to ϵ_A and ϵ_B. The main difference of the dative bond compared to the ordinary covalent bond is that one of the orbitals ($|\Phi_A\rangle$ or $|\Phi_B\rangle$) is initially fully occupied, and the other is initially fully unoccupied, as shown in Fig. S3. Again, the system can reduce its energy by sharing both electrons in the bonding state $|\Psi\rangle$ which is still just a proper linear combination of similarly contributing states $|\Phi_A\rangle$ and $|\Phi_B\rangle$. Therefore, again the polarity p according to Eqn. (2) is small for the dative bond. However, since both electrons are donated from only one component towards a bonding state $|\Psi\rangle$, that is shared between both components A and B, there is a nominal charge transfer from the donor towards the acceptor,

resulting in a net dipole between A and B in the case of a dative bond.

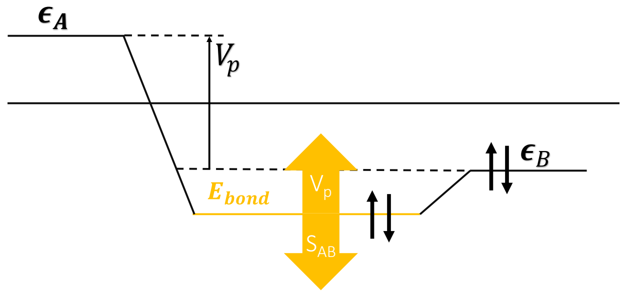

Figure S3: Illustration of the occupation in a dative bond.

With increasing energy difference between $|\Phi_A\rangle$ and $|\Phi_B\rangle$, also the imbalance between the contributions $|c_A|$ and $|c_B|$ to the bond state $|\Psi\rangle$ increases. The ionic bond represents the most extreme case in which the energy difference $V_p = |\epsilon_A - \epsilon_B|$ is so large that $|c_A|$ and $|c_B|$ are either 0 and 1 or vice versa. This means that the bonding state $|\Psi\rangle$ is represented only by one of the states $|\Phi_A\rangle$ or $|\Phi_B\rangle$, namely the one with the lower energy. If both states $|\Phi_A\rangle$ and $|\Phi_B\rangle$ initially hold one electron, the system can still decrease its energy by transferring one electron from the energetically higher state towards the energetically lower lying state, but this energetic gain is not related to the formation of a hybrid orbital as the linear combination of $|\Phi_A\rangle$ and $|\Phi_B\rangle$. Rather, the energy reduction only results from the large energy difference between the two states $|\Phi_A\rangle$ and $|\Phi_B\rangle$. In the case of an ionic bond, the polarity p is large (i.e., 1 in the most extreme case) because $|c_A|$ and $|c_B|$ are either 0 and 1 or vice versa. In contrast to the case of a dative bond, here both the polarity p is large (i.e., 1 in the most extreme case) and the nominal charge transfer is large because one electron is transferred from one component to the other (compare Fig. S4).

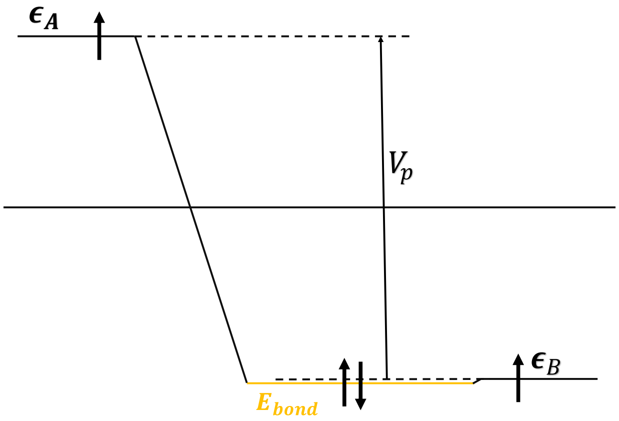

Figure S4: Illustration of the energy and occupation for a typical ionic bond.

Summarising, the polarity p can distinguish whether the character of a bond is more ionic or covalent/dative. However, as described above, it cannot directly yield the amount of charge transfer, i.e., the correlated dipole between the components A and B, because both dative and ionic bonds yield a large charge transfer but contrary values for the polarity p. To make statements about the charge transfer, i.e., the resulting dipole between the components A and B, both the polarity p and the initial occupation of the involved states $|\Phi_A\rangle$ and $|\Phi_B\rangle$ need to be known. Figure S5 a) illustrates how the charge transfer evolves with respect to the polarity p for the discussed bond archetypes.

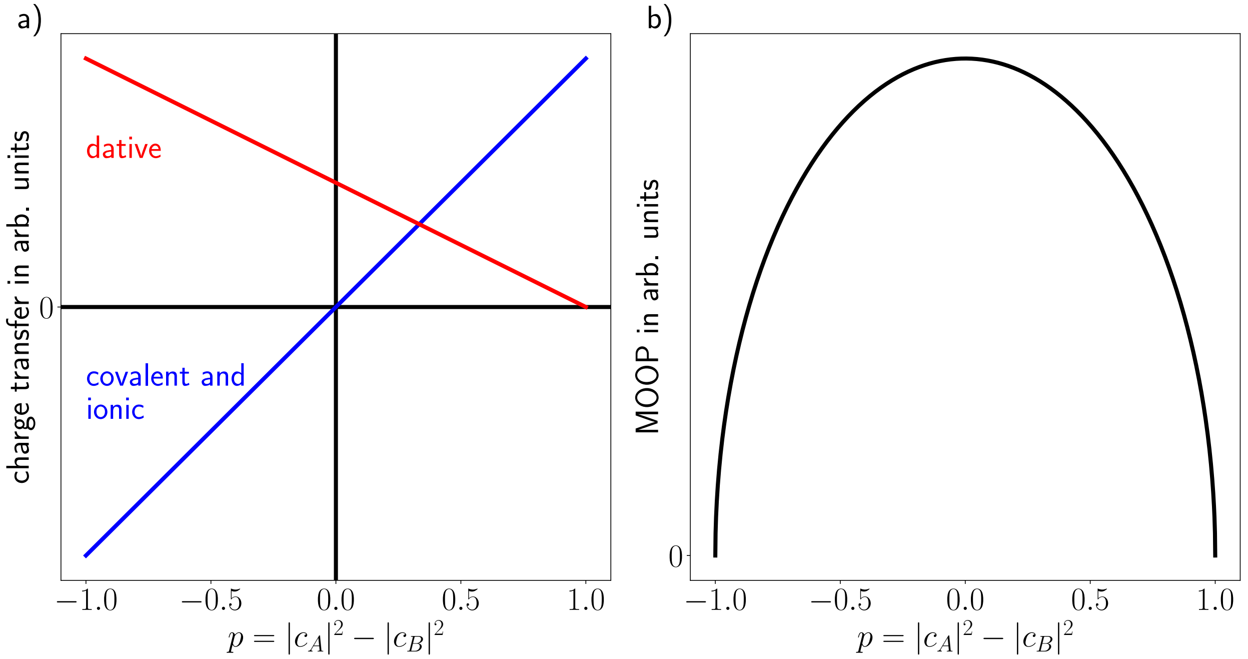

Figure S5: a) Charge transfer between both bonding partners depending on the polarity for the discussed bond archetypes. b) Dependence of the MOOP on the polarity .

The polarity p can be estimated from the MOOP, which itself is proportional to $S_{AB}c_A^*c_B$. Figure S5 b) shows how the MOOP scales with the polarity p. To discriminate between ordinary covalent bonds and dative bonds, one can make use of the MOPDOS. For example, a broad peak below the Fermi level in the MOPDOS that can be associated with a former LUMO of the adsorbed molecule yields the conclusion that the bond has a strong dative character, if in this case also the MOOP is large. This is because the broadening of the peak

suggests hybridisation between the involved components (here the molecule's LUMO and the substrate surface states) and the fact the LUMO was, obviously, initially unoccupied.

S3 Atomic overlap population

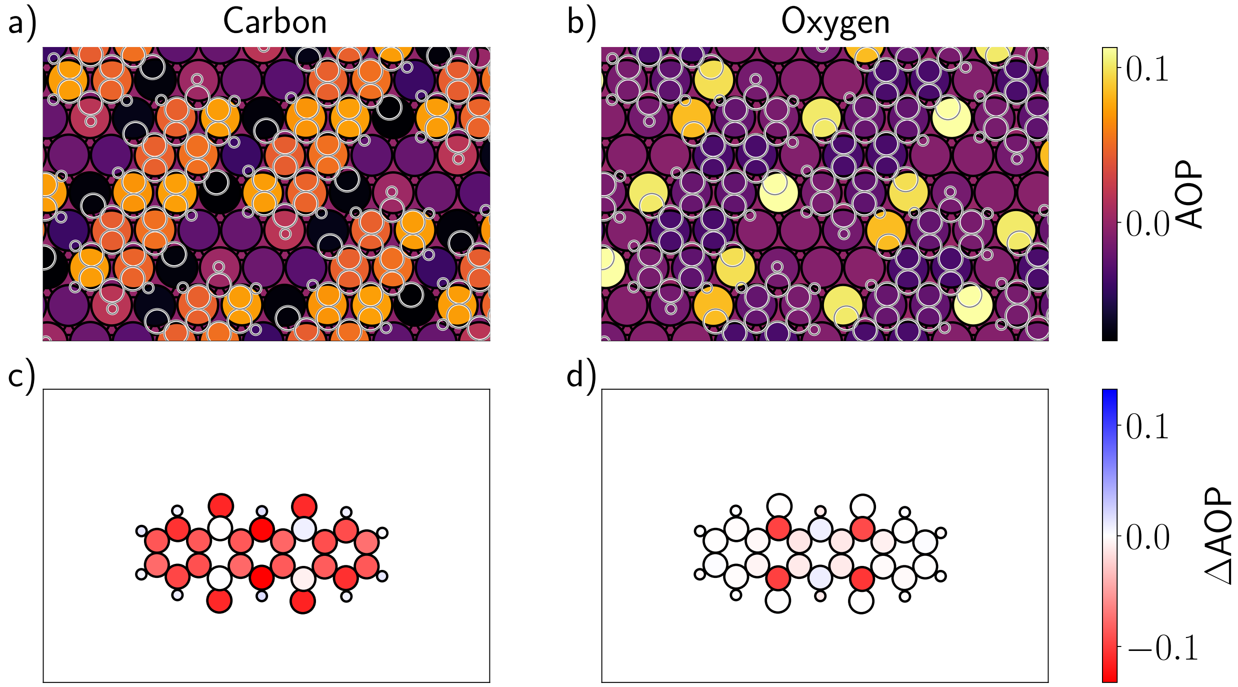

Figure S6: The atomic overlap population (AOP) for different atomic species. The AOP is the overlap population for the entire occupied region between two atoms A and B. It yields insights into the amount of charge which is shared between A and B and is directly connected to the bond order between A and B. In the plot the AOP for a specific species is shown which corresponds to the sum of all atoms A (which are of this species) and one specific atom B. a) The AOP between all C atoms (atoms A) and each Cu atom (atom B). The molecule is drawn as outline to illustrate the adsorption sites of the individual atoms. The high overlap population between the C atoms and the surface is a manifestation of the discussed dative bond. b) The same as in a) but for the AOP between O and Cu, again showing a bonding character towards the surface. c) The intramolecular changes in the AOP between the C atoms and all molecular atoms upon adsorption on the Cu(111) surface. d) The same as in c) but for the O atoms. c) and d) indicate the loss of overlap population in the molecule. Especially the AOP between C and O decreases indicating a reduction in bond order. We point out that this reduction, however, does not result in an overlap population which would be expected for a single bond between C and O, as it would be the case for an sp^3 hybrid.

S4 Complete simulation of the photoelectron momentum maps

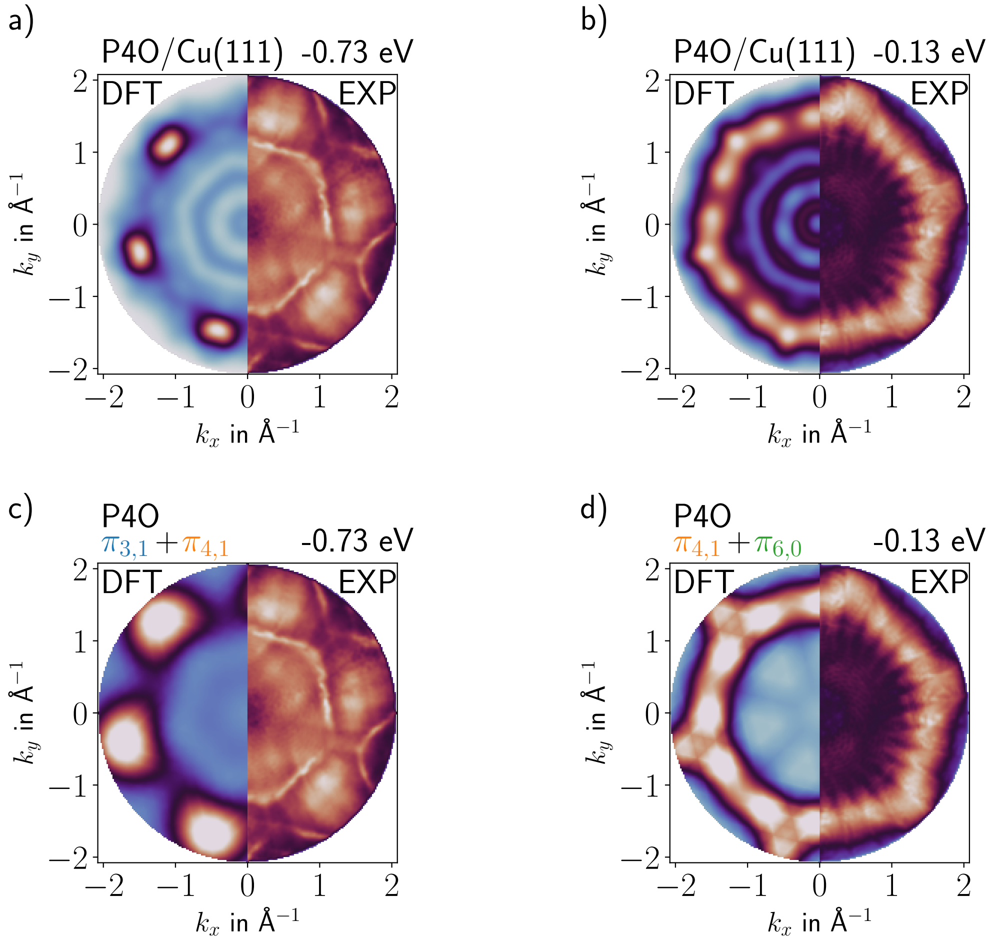

Figure S7: Simulated and measured photoelectron momentum maps of P4O on Cu(111). The left half of each map is the simulation. The corresponding right half shows the measured intensity distribution. In all cases the simulation is scaled to the intensity maximum of the measurement. All symmetries of the substrate are taken into account for the simulation. The top simulations of a) and b) are done using the entire system of P4O on Cu(111), while the simulations of c) and d) are done using the gas-phase P4O molecule. Maps a) and c) are taken at a binding energy of -0.73 eV and correspond to the intensity distribution of the $\pi_{3,1}$ and $\pi_{4,1}$ orbital emissions; b) and d) are taken at a binding energy of -0.13 eV and show emission from the $\pi_{4,1}$ and $\pi_{6,0}$ orbitals.

To verify that both electronic configurations obtained from different DFT implementations are valid, the MOPDOS are compared to each other in Fig. S8. Note that for the MOPDOS from FHI-aims, which is also shown in the main text, the employed sampling and smoothing differ from the MOPDOS obtained from VASP.

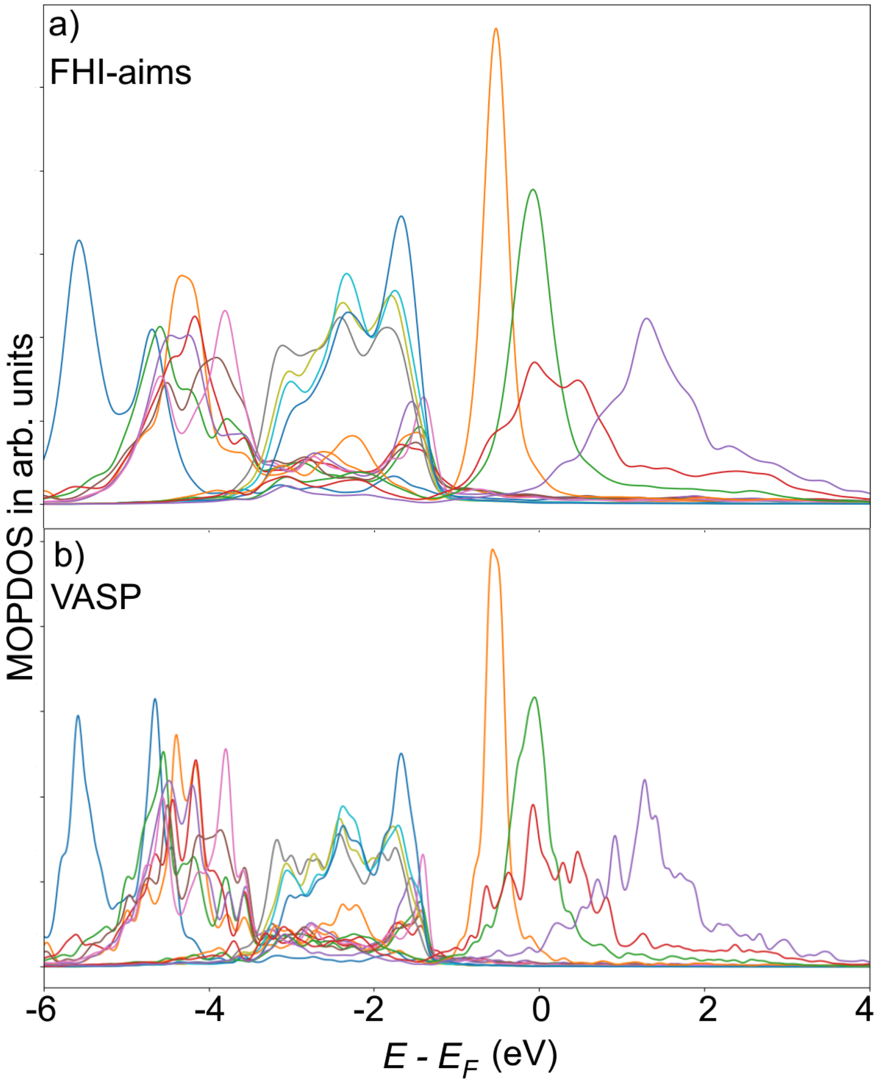

Figure S8: Validation of the MOPDOS obtained from different DFT calculations. a) The MOPDOS from FHI-aims which is also shown in the main text. b) MOPDOS as obtained from VASP which is used for the simulation of the full momentum maps in the main text. Note that the VASP MOPDOS was done with less k-smoothing.

S5 Similarity measure of aromaticity

To connect the π-system occupation to the aromaticity on a surface we developed a model that relates the observed changes in the π-electron system on the surface to a reference aromaticity in a quantitative manner.

Firstly, a connection to a parent aromatic hydrocarbon is established. Then the reference aromaticity is defined as the aromaticity of the chosen compound. In general, any structural analogue with a higher degree of aromaticity can be chosen as aromatic parent molecule. The following model then represents a "similarity"-measure, quantifying how similar the π-system of the molecule on a surface is compared to the π-system of the chosen aromatic parent.

For P4O the aromatic parent pentacene (PEN) is a straightforward choice. Being an acene, PEN is a prototypical aromatic compound with a fully conjugated π-system, which delocalises over the entire molecular backbone. Therefore, if P4O was to match the π-system of PEN, both would resemble a molecule with the same degree of aromaticity. In the following we scrutinise this connection between P4O and PEN.

When the π-orbitals of the gas-phase P4O are compared to those of gas-phase PEN it becomes obvious that both systems show the same orbital symmetries with an offset in the occupations. The highest occupied π-orbital of gas-phase P4O corresponds to the third highest occupied of PEN. This connection is also illustrated in the main text Fig. 2 d). A general expression for this offset (from now on referred to as N_e^π) can be found using Eqn. (3), in which the π-orbital occupations f_i^π are summed for the molecule and the chosen parent compound in the gas phase. N_e^π, thus, denotes how different the number of π-electrons of the two molecules is, which represents a first measure of a "distance" between the molecules.

$$N_e^\pi = \left| \sum_{i=0}^{\infty} f_i^{\pi\text{-mol}} - \sum_{i=0}^{\infty} f_i^{\pi\text{-parent}} \right| \tag{3}$$

For P4O relative to PEN (both in the gas phase) N_e^π amounts to 4. Now, if upon adsorption

on a surface the occupations of the π-system changes, these changes will also affect the "distance" to the chosen parent. If the occupations change in a way that now they match the π-system occupation of the chosen parent molecule in the gas phase, the gas-phase "distance" N_e^π has vanished. This would correspond to the closest possible "distance" and the aromaticity of the described molecule would be equivalent to that of the reference aromaticity.

For P4O this would be the case if the LUMO ($\pi_{3,1}$) and LUMO+1 ($\pi_{4,1}$) become fully occupied on the surface. However, if the charge transfer results in the occupation of even higher π-orbitals (for P4O the $\pi_{6,0}$-orbital and beyond) the π-system on the surface deviates from the parent molecule and the "distance" increases. Combining these conditions leads to Eqn. (4), in which N_e^π corresponds to the already discussed gas-phase offset, and N represents the total number of electrons in the gas phase (therefore $N/2$ corresponds to the HOMO in the gas phase). The equation yields the quantity A, which we interpret as a measure for the aromatic "distance" between the gas-phase parent and the molecule of interest adsorbed on the substrate.

$$A = N_e^\pi - \sum_{i>N/2}^{N/2+N_e^\pi/2} f_i^{\pi\text{mol-surf}} + \sum_{i>N/2+N_e^\pi/2}^{\infty} f_i^{\pi\text{mol-surf}} \tag{4}$$

The value A has a lower bound of 0 which corresponds to the case in which both π-systems match. If A is lower than N_e^π the differences between the molecule and the chosen parent become smaller on the surface, and if A is larger than N_e^π the discussed difference increases. To enable a better comparison between different systems, A can be normalised to N_e^π, yielding the normalised aromaticity similarity A_{sim} according to Eqn. (5). This is used in the main text and in section S10.

$$A_{\text{sim}} = 1 - A/N_e^\pi \tag{5}$$

Therefore, A_{sim} is a "similarity" measure for the π-system of an adsorbed molecule and a

chosen parent in the gas phase. This approach also allows for the parent molecule to be equal to the molecule of interest. In this case N_e^π would be zero (i.e., there is obviously no difference for the exact same π-system in the gas phase) and Eqn. (4) reduces to the last sum. In this special case the "similarity" can only decrease upon adsorption on a surface (it was perfectly matching in the gas phase already and can only deviate from that). Because a normalisation is ill defined for $N_e^\pi = 0$ it can be chosen arbitrarily, however if the obtained reference aromaticity is to be compared with other systems a common normalisation should be chosen. This ensures that the same change in some π-system occupation δf results in the same change in the normalised aromaticity similarity δA_{sim} between the compared systems.

S6 Distance dependence

To understand the dependence of the aromaticity similarity δA_{sim} on the adsorption height d between the molecule and the surface, a simple model is given for the purely ionic case. If only purely ionic interactions are present, no broadening and no reordering of the molecular states occur (compare section S2). This means that transferred charge obeys the gas-phase ordering of the molecular states.

Therefore, the transferred charge ΔQ is only dependent on the potential difference between the molecule and the substrate (i.e., the position of HOMO/LUMO in the gas phase and the Pauli-pushback reduced work function of the surface as illustrated in Fig. S9[S2]), as well as the area of the molecule A_{mol} and the distance (real space) to the mirror plane d according to Eqn. (6).[S3]

$$\Delta\Phi_q = -\frac{q_e}{\epsilon_0}\frac{\Delta Q \cdot d}{A_{\mathrm{mol}}} \tag{6}$$

Thus, the amount of charge transferred (corresponding to the occupation of the respective state which is filled/emptied) is proportional to one over the real space distance d. Because the aromaticity similarity A_{sim} scales linearly with the change in the π-system occupation it will therefore also scale with one over the adsorption height for purely ionic interactions.

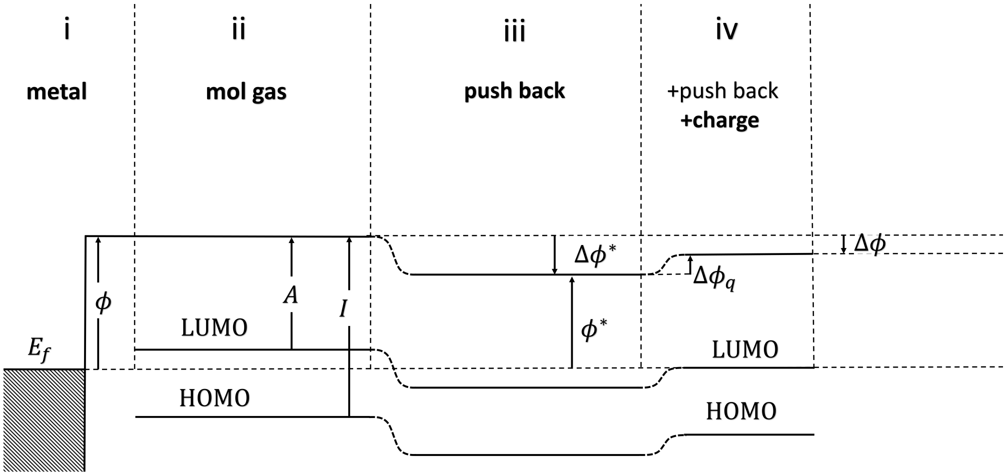

Figure S9: Simplified energy level alignment for the metal surface (i) with work function Φ and a molecule in the gas phase (ii). Section iii shows the reduction of the work function $\Delta\Phi^*$ caused by the Pauli repulsion at the adsorbate-substrate interface. Section iv shows the work function modification $\Delta\Phi_q$ caused by the charge transfer between the substrate and the adsorbate. Based on the work of Ishii et al.[S2]

S7 Experimental data of P4O on Ag(111)

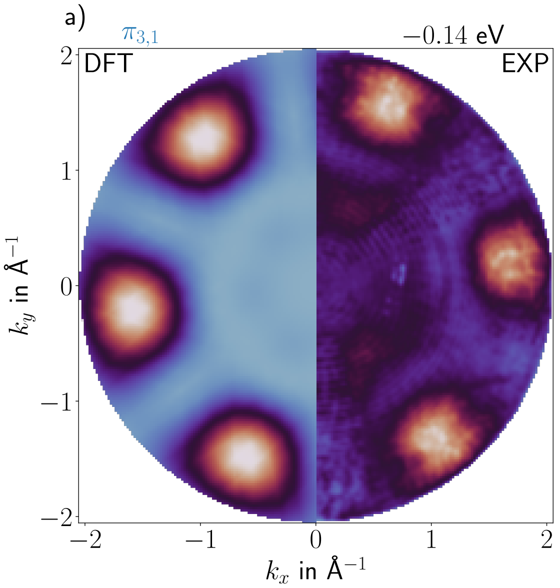

Figure S10: Measured photoelectron momentum map of P4O on Ag(111) taken at a binding energy of $-0.14\,\text{eV}$. The left half shows the simulation, which is done using the $\pi_{3,1}$-orbital of the gas-phase P4O molecule.

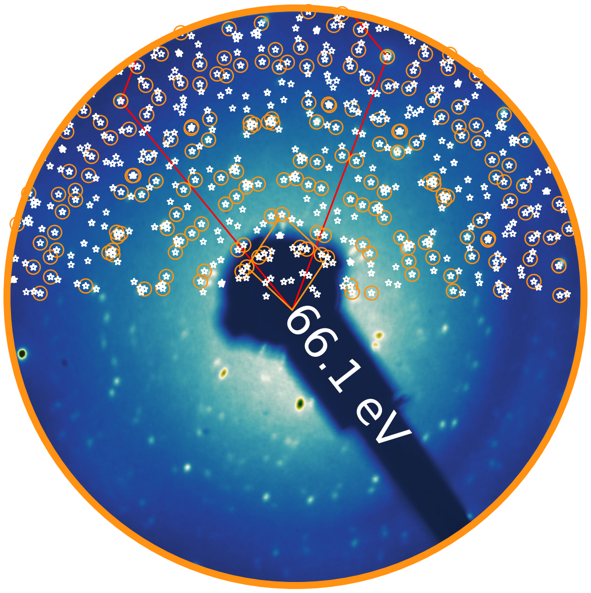

Figure S11: Distortion-corrected LEED image of P4O on Ag(111) taken at a primary energy of 66.1 eV. The red spots correspond to the Ag(111) lattice. In orange the primary spots of the P4O lattice are shown, along with spots resulting from multiple scattering as smaller white stars.

Using a fitting procedure we find an epitaxial matrix describing the relation between P4O and the Ag(111) surface as $\begin{pmatrix} 4.00(1) & 0.54(2) \\ 1.00(1) & 4.19(1) \end{pmatrix}$. The substrate lattice at 300 K is given by $|\vec{s}_1| = |\vec{s}_2| = 2.8898\,\text{Å}$ and $\measuredangle(\vec{s}_1, \vec{s}_2) = 120°$.[S4]

S8 Distance sweeps for P4O on Cu(111) and on Ag(111)

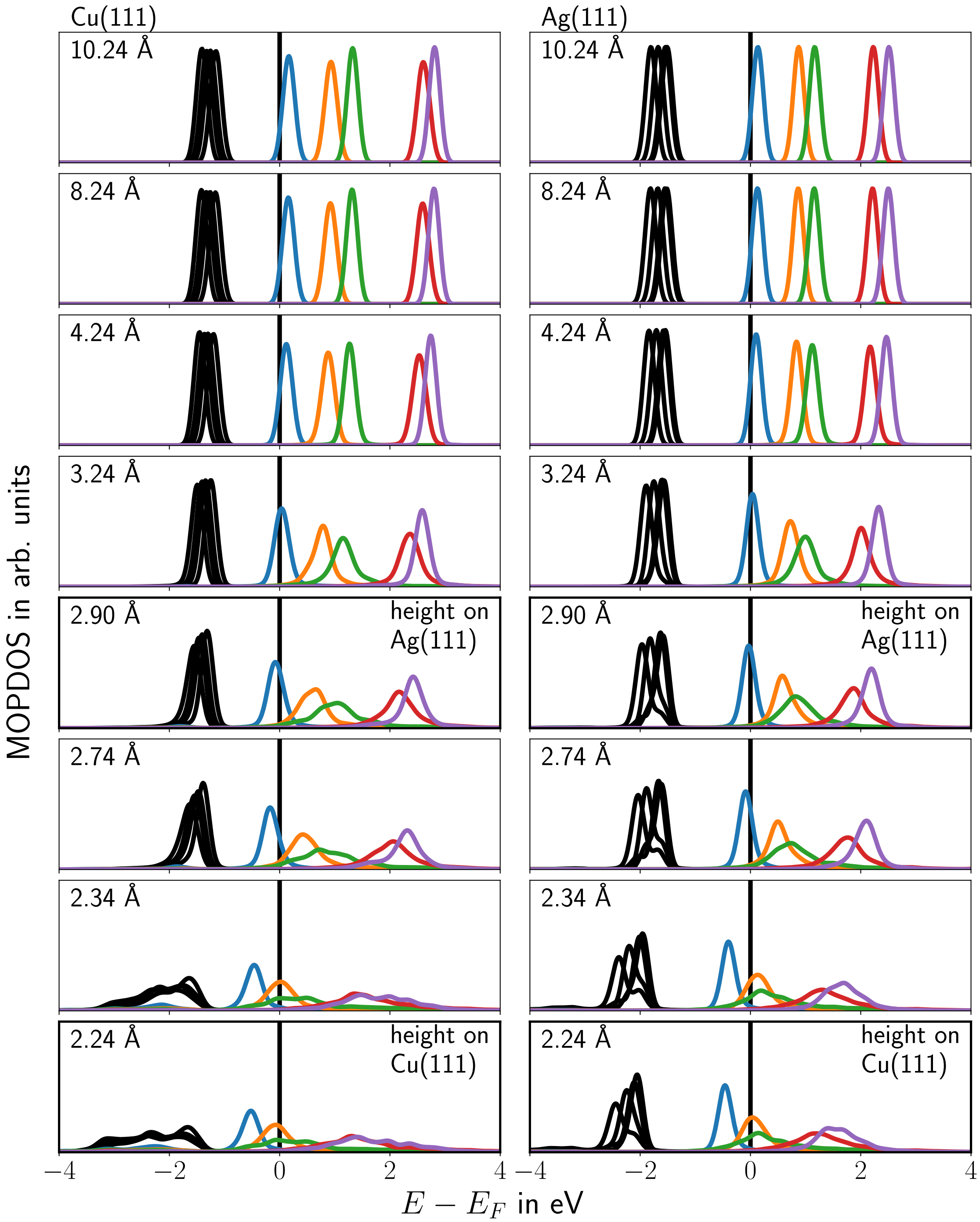

Figure S12: Distance sweeps for the systems P4O on Cu(111) (left) and on Ag(111) (right). The molecules were positioned at equal distances from the surface (average carbon plane to average top most metal atom plane). The optimal adsorption heights for P4O on Cu(111) and Ag(111) are annotated accordingly. For each distance the MOPDOS was calculated (see main text for more details).

S9 PEN on Cu(111)

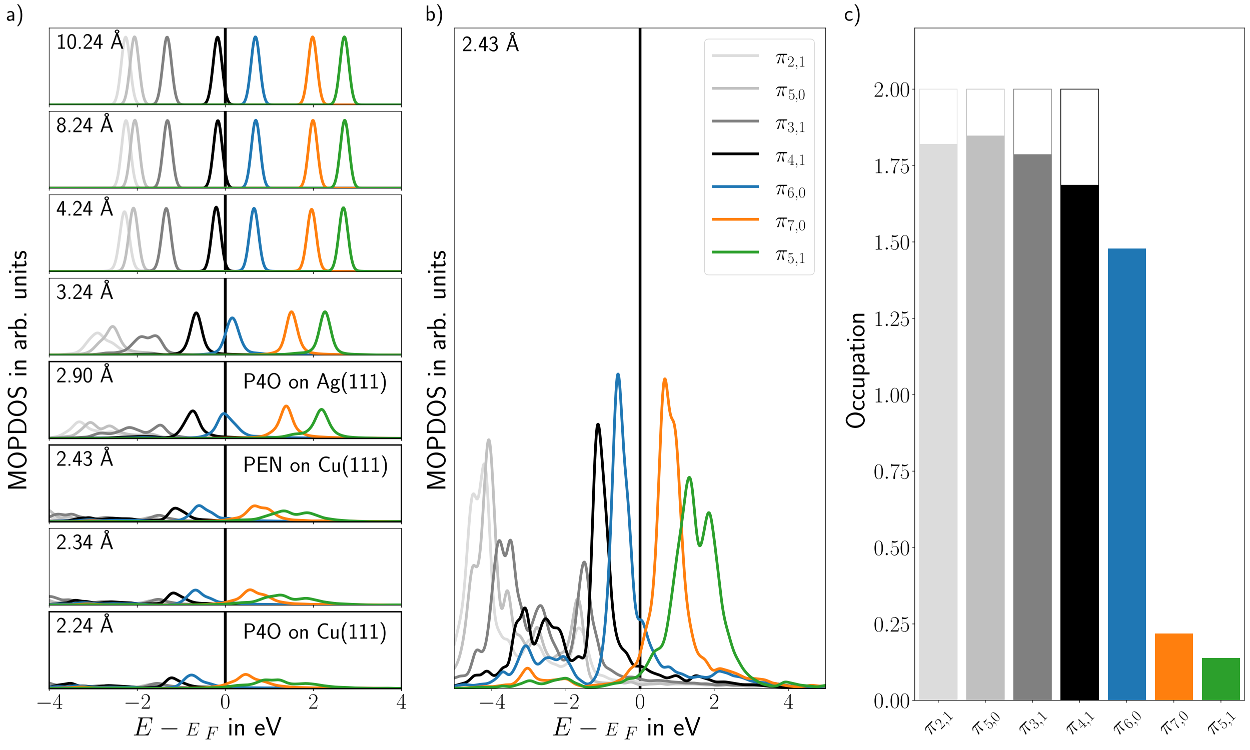

Figure S13: a) MOPDOS for different hypothetical adsorption heights of PEN on Cu(111). The adsorption heights of P4O on Cu(111) and Ag(111) are indicated accordingly. This distance sweep in conjunction with the quantification of the aromaticity (cf. section S5) was used in the aromaticity over distance plot in Fig. 5 c) of the main text. b) MOPDOS of PEN on Cu(111) for the energetic minimum. The molecular π-system hybridises with the Cu(111) surface, resulting in a large energetic broadening and an occupied LUMO. The occupations for the energetic minimum can be seen in c). The outlined parts correspond to the occupation of PEN in the gas phase.

S10 Further investigated systems

To further investigate the mechanism in which dative bonds may dominate over ionic interactions on a surface, we focus on two different molecules which should undergo an aromatic stabilisation on the surface. The first molecule is 4,5,9,10-pyrenetetrone (Py4O) of which the π-system is expected to convert into a pyrene-like molecule when it undergoes an aromatic stabilisation. The adsorption structure of Py4O as obtained from DFT can be seen in Fig. S14 a) for Py4O on Cu(111) and in b) on Ag(111). For both systems the LUMO and LUMO+1 are energetically close together and (partially) below the Fermi level (compare Fig. S14 d) and e)). The resulting occupations (see Fig. S14 g) and h)) show that indeed both π-orbitals receive charge. However, in none of both cases the electronic situation of gas-phase pyrene is reached. Nonetheless, Py4O on Cu(111) comes close with a similarity to the reference aromaticity of $A_{\mathrm{sim}} = 0.74$ which is the highest value we found for all investigated systems. Similarly to P4O (compare main text) the interaction on Ag(111) is weaker and the similarity to the reference aromaticity is smaller with a value of $A_{\mathrm{sim}} = 0.50$. When examining the MOOP (compare Fig. S14 j) and k)) for both systems we again find that the formerly unoccupied π-orbitals form dative bonds with the surface. Especially Py4O on Cu(111) shows high bonding contributions towards Cu(111) at around $-2.5\,\mathrm{eV}$.

The last molecule we investigated is 1,2,5,6,9,10-coronenehexone (Cor6O) on Cu(111). Upon a complete aromatic stabilisation Cor6O would be converted to a coronene-like molecule. The lateral structure on Cu(111) is shown in Fig. S14 c). For Cor6O the LUMO, LUMO+1, and LUMO+2 are below the Fermi level showing a similar energetic positioning (with respect to the Fermi level) when compared to P4O on Cu(111) (compare main text), with the important difference that in coronene the LUMO+2 would be fully occupied in the gas phase (unlike the case of P4O and PEN). The corresponding occupations are shown in Fig. S14 i) and reveal significant charge in the aforementioned orbitals. However, the electronic situation of coronene in the gas phase is not reached. We find a similarity to the reference aromaticity of $A_{\mathrm{sim}} = 0.70$ which is the second highest found in our study. Lastly, the MOOP

(Fig. S14 l)) again shows the mechanism of dative bonds with the surface, having strong bonding features at around $-2.5\,\mathrm{eV}$. We conclude that this mechanism of dative bonds dominating the interaction with the surface is not limited to the system of P4O and likely occurs in other such strongly hybridised systems.

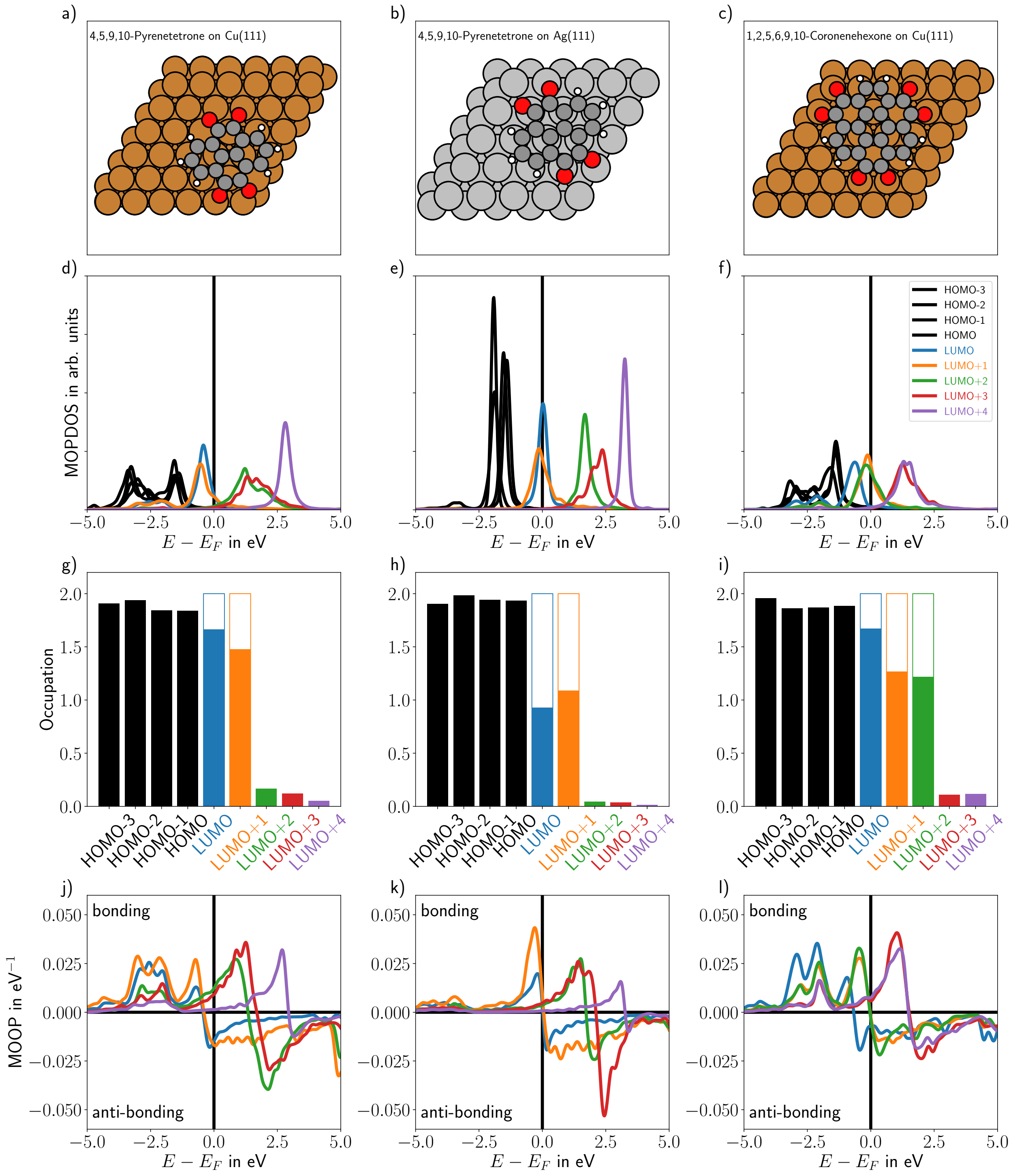

Figure S14: Further investigated systems which all show an aromaticity similarity $A_{\text{sim}} < 1$ and dative bonds with the surface. Same columns always correspond to the same system: a) 4,5,9,10-pyrenetetrone (Py4O) on Cu(111), b) Py4O on Ag(111), and c) 1,2,5,6,9,10-coronenehexone (Cor6O) on Cu(111). The colours of the circles correspond to the chemical elements: dark orange - Cu, light grey - Ag, red - O, dark grey - C, and white - H. d), e), f) are the corresponding MOPDOSs; g), h), i) display the resulting occupations; j), k), l) depict the corresponding MOOPs.

References

(S1) Lu, X.-G.; Chen, Q. A CALPHAD Helmholtz energy approach to calculate thermodynamic and thermophysical properties of fcc Cu. *Philos. Mag.* **2009**, *89*, 2167–2194.

(S2) Ishii, H.; Sugiyama, K.; Ito, E.; Seki, K. Energy Level Alignment and Interfacial Electronic Structures at Organic/Metal and Organic/Organic Interfaces. *Adv. Mater.* **1999**, *11*, 605–625.

(S3) Zojer, E.; Taucher, T. C.; Hofmann, O. T. The Impact of Dipolar Layers on the Electronic Properties of Organic/Inorganic Hybrid Interfaces. *Adv. Mater. Interfaces* **2019**, *6*, 1900581.

(S4) Guo, X.; Xu, Z.-F.; Lu, X.-G. A CALPHAD Helmholtz Energy Approach to Assess the Thermodynamic and Thermophysical Properties of fcc Ag. 2nd Annual International Conference on Advanced Material Engineering (AME 2016). 2016.
